# Biological Properties of Acidic Cosmetic Water from Seawater

**DOI:** 10.3390/ijms13055952

**Published:** 2012-05-16

**Authors:** Wei-Ting Liao, Tsi-Shu Huang, Chien-Chih Chiu, Jian-Liang Pan, Shih-Shin Liang, Bing-Hung Chen, Shi-Hui Chen, Po-Len Liu, Hui-Chun Wang, Zhi-Hong Wen, Hui-Min Wang, Shu-Wen Hsiao

**Affiliations:** 1 Department of Biotechnology, Kaohsiung Medical University, 100, Shih-Chuan 1st Road, San-Ming District, Kaohsiung 807, Taiwan; E-Mails: wtliao@kmu.edu.tw (W.-T.L.); cchiu@kmu.edu.tw (C.-C.C.); l3893117@gmail.com (S.-S.L.); bhchen@kmu.edu.tw (B.-H.C.); 2 Section of Microbiology, Department of Pathology and Laboratory Medicine, Kaohsiung Veterans General Hospital, Kaohsiung 807, Taiwan; E-Mail: tsishuhuang@gmail.com; 3 Department of Medical Technology, Fooyin University, Kaohsiung County 831, Taiwan; 4 School of Medicine, National Yang-Ming University, Taipei 112, Taiwan; 5 Department of Chemical and Biochemical Engineering, Kao Yuan University, Kaohsiung 821, Taiwan; E-Mail: t50050@cc.kyu.edu.tw; 6 Department of Research and Development, Taiyen Biotech Co., Ltd., 15, Gong-Huan Road, Annan District, Tainan 709, Taiwan; E-Mail: tsi150@tybio.com.tw; 7 Department of Respiratory Therapy, College of Medicine, Kaohsiung Medical University, 100, Shih-Chuan 1st Road, San-Ming District, Kaohsiung 807, Taiwan; E-Mail: kisa@kmu.edu.tw; 8 Graduate Institute of Natural Products, College of Pharmacy, Kaohsiung Medical University, Kaohsiung 807, Taiwan; E-Mail: wanghc@kmu.edu.tw; 9 Department of Marine Biotechnology and Resources, National Sun Yat-sen University, 70, Lien-Hai Rd, Kaohsiung 804, Taiwan; E-Mail: wzh@mail.nsysu.edu.tw; 10 Department of Fragrance and Cosmetic Science, Kaohsiung Medical University, 100, Shih-Chuan 1st Road, San-Ming District, Kaohsiung 807, Taiwan

**Keywords:** acidic cosmetic water (ACW), antioxidant activity, anti-microorganism, anti-inflammation, allergy-free, skin-whitening, anti-melanoma

## Abstract

This current work was to investigate the biological effects of acidic cosmetic water (ACW) on various biological assays. ACW was isolated from seawater and demonstrated several bio-functions at various concentration ranges. ACW showed a satisfactory effect against *Staphylococcus aureus*, which reduced 90% of bacterial growth after a 5-second exposure. We used cultured human peripheral blood mononuclear cells (PBMCs) to test the properties of ACW in inflammatory cytokine release, and it did not induce inflammatory cytokine release from un-stimulated, normal PBMCs. However, ACW was able to inhibit bacterial lipopolysaccharide (LPS)-induced inflammatory cytokine TNF-α released from PBMCs, showing an anti-inflammation potential. Furthermore, ACW did not stimulate the rat basophilic leukemia cell (RBL-2H3) related allergy response on de-granulation. Our data presented ACW with a strong anti-oxidative ability in a superoxide anion radical scavenging assay. In mass spectrometry information, magnesium and zinc ions demonstrated bio-functional detections for anti-inflammation as well as other metal ions such as potassium and calcium were observed. ACW also had minor tyrosinase and melanin decreasing activities in human epidermal melanocytes (HEMn-MP) without apparent cytotoxicity. In addition, the cell proliferation assay illustrated anti-growth and anti-migration effects of ACW on human skin melanoma cells (A375.S2) indicating that it exerted the anti-cancer potential against skin cancer. The results obtained from biological assays showed that ACW possessed multiple bioactivities, including anti-microorganism, anti-inflammation, allergy-free, antioxidant, anti-melanin and anticancer properties. To our knowledge, this was the first report presenting these bioactivities on ACW.

## 1. Introduction

First of this present study was designed to evaluate the antibacterial activity against representative gram-positive and gram-negative strains that were frequently encountered in the hospital environment. Electrolysis water might be a powerful bactericidal agent which exhibited significant effects on inhibiting bacteria growth even during a short immersion time [1]. Moreover, it needs to be established that it does not harm to the human body [2]. Therefore, the candidate agent was evaluated for use as a disinfectant for waterline and medical devices. Previous studies showed that dental unit waterline [3] and ocular surfaces [4] could be sterilized by acidic electrolyzed water.

Continuing this current work, we further detected immune responses and the way these are regulated. An important component of the immune system is the T-helper (Th) cell that initiates and regulates immune responses [5]. There are 4 various major subtypes of Th cells. Th1 regulates inflammatory response against infections, while Th2 modulates allergic responses. Regulatory Th (Treg) plays a pivotal role in immune suppression. Th17 is associated with autoimmunity. Among them, the inflammatory responses of Th1 are primarily triggered by cytokines from monocytes/macrophages. When monocytes polarize to macrophages, they recognize antigens and secrete pro-inflammatory cytokines, such as TNF-α and interleukins (ILs). TNF-α and IL-6 released from monocytes/macrophages further encounter and activate antigen-specific T cell killing, *i.e.*, the Th1 responses. In contrast, macrophages also alternatively secrete Th2 cytokines, such as IL-4, under allergen exposure and activate Th2-mediated allergic responses [6].

Given the negative feedback nature between Th1 and Th2 (activating one will inactivate the other), an agent with an anti-inflammatory effect may also exhibit, in parallel, an increased allergic effect. The physiological consequences of type I allergic diseases are mainly highlighted by immunoglobulin E (IgE)-mediated activation of mast cells or basophils. Mast cells are widely distributed throughout mucosal and connective tissues. They are considered the pivotal cell type in defending against parasites and bacteria in innate immunity, and on the other hand, they are also the major effector cells of IgE-mediated allergic inflammation [7]. Allergen-specific IgE is directly involved in the initiation of allergic cascade by binding to the high-affinity receptors for IgE (FcɛRI), expressed on the surface of either mast cells or basophils. Re-exposure to the same allergen will cause the cross-linking of FcɛRI-bound IgE molecules and trigger downstream signaling events, finally resulting in the elevation of intracellular calcium levels for mast cells to degranulate various inflammatory mediators.

Aside from anti-bacterial and immune responses, several additional biological effects were tested in the present study, including anti-oxidation, anti-pigmentation, and anti-tumor activities. Agents with antioxidant properties are well recognized in their prevention of some diseases. Over the past decade, interest in anti-oxidative properties has increased markedly, due to related protective properties that combat various diseases [8]. Anti-oxidative constituents are crucial in cosmetic products, due to their ability to reduce free radical-mediated degradation of skin and related organs in human beings. The mechanism generally accepted is that free radical-scavenging activities reduce oxidative stress and thus prevent disease [9].

Hyper-pigmentations, such as senile lentigo, melasma, freckles and pigmented acne scars, are of particular concern to women, and even men [10]. Their treatment usually involves the use of medicines or medicinal cosmetics containing de-pigmenting agents or skin-whitening agents. However, issues on safety mean that only a few effective natural and synthetic regulators that act to minimize skin pigmentation abnormalities are used as therapeutic and cosmetics agents. Recently, much attention has been directed by medical practitioners and cosmetics businesses to the major application strategies of tyrosinase inhibitors for the treatment of hyper-pigmentation [11]. Tyrosinase catalyzes two distinctly significant reactions in melanin synthesis: the hydroxylation of L-tyrosine to 3,4-dihydroxy-L-phenylalanine (L-dopa) and the oxidation of L-dopa to dopaquinone, followed by further conversion to melanin production. In the clinical setting, tyrosinase inhibitors are used to treat dermatological disorders related to melanin hyper-accumulation, and are thus essential in cosmetics for de-pigmentation.

Human skin is frequently affected by oxidative stress which is produced by intrinsic and external sources induced by free radicals and ultraviolet (UV) radiation [12]. There have been study reports that skin exposed to oxidative stress or UV is linked to photoaging and tumor generation [9,12–14]. Solar UV exposure is associated with over two-thirds of the cases and may be a major etiological factor in skin cancer [13]. Occurrences of cutaneous malignant melanomas have increased in the past several decades. Since it has a strong propensity to metastasize, it is one of the most aggressive illnesses among skin cancers [14]. Unlike other cancers, metastatic melanoma is not easy to treat by the standard methods of surgery, radiotherapy or chemotherapy [15]. Therefore, metastatic melanoma frequently leads to poor patient prognosis [16]. A naturally occurring agent that can induce cancer cell death without significant side effects would be a good chemopreventive agent.

Recently, acidic water has been applied in medicine. Acidic cosmetic water (ACW) is a colorless, transparent solution prepared by the electrolysis of water with electrolytes. A previous report has documented several advantages of ACW, including: harmless to the environment; economical; anti-allergenic and toxicity free. After the breakdown of ACW, the final products are limited to saline and traces of chlorine gas. The production price of ACW is cheap—just water and electrolyte. Since the basic elements of ACW are ions and water, there is no concern about allergic reaction. Besides that, ACW is a weak acid solution showing no extra cytotoxicity [17], and the bio-functions of ACW were being tested for medical treatments [18]. Despite that, deep sea water was reported to exert many biological effects on cells, including anti-virus [19], anti-diabetes [20], improving the cardiovascular hemodynamics [21] and anti-hyperlipidemia [18].

## 2. Materials and Methods

### 2.1. Reagents and Materials

Vitamin C, dimethyl sulfoxide (DMSO), 1,1-diphenyl-2-picrylhydrazyl (DPPH), ethylene diamine tetra-acetic acid (EDTA), 2,6-di-*tert*-butyl-4-methylphenol (BHT), nitroblue tetrazolium (NBT), 2,2′-azino-bis-(3-ethylbenzothiazoline-6-sulphonic acid) (ABTS), 2,4,6-tripyridyl-*S*-triazine (TPTZ), phenazine methosulfate (PMS), nicotinamide adenine dinucleotide (NADH), potassium ferricyanide (K_3_Fe(CN)_6_), trichloroacetic acid, FeCl_3_ and FeCl_2_·4H_2_O were purchased from Sigma-Aldrich Chemical (St. Louis, MO, USA). All buffers and other reagents were of the highest purity commercially available.

### 2.2. Preparation of ACW from Seawater

**Figure 1:**
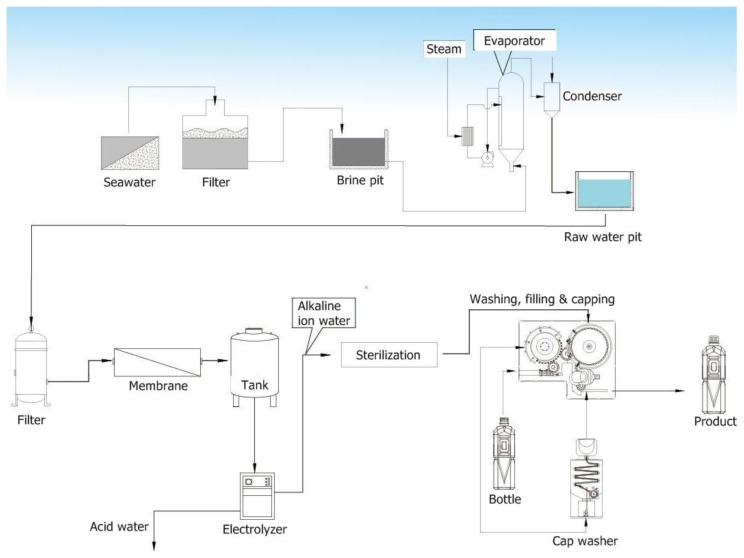
The protocol of acidic cosmetic water (ACW) purified processes.

Seawater was separated into water, crystal salt and bittern by electro-dialysis followed by evaporation crystallization. It was collected in intake systems, passed through a sand filter, ion-exchange electrodialysis, brine pit and evaporator systems to get condensed water. The condensed water was then passed through a 1 μm filter, reverse osmosis membrane and electrolyzed to get acidic water with edible electrolyte added to the water. The conductivity of ACW is about 199.6 ± 2.1 S/cm (Figure 1). ACW was prepared and stored at 4 °C in opaque containers.

### 2.3. Determination of Antimicrobial Activity

*Staphylococcus aureus* (ATCC 29213) and *Escherichia coli* (ATCC 35218) were harvested in normal saline and adjusted to McFarland 0.5 (1.5 × 10^8^ CFU/mL). One milliliter samples of the bacterial suspension were centrifuged at 9000 rpm for 3 min, and treated with 0.95 mL of either ACW or saline control for reactions of 5, 30, 60, 180, 300, 900 s. The well, which contains saline, serves as growth control. A negative control, which contains acidic water only, and a reagent control which contains medium only, were included in each test. Following the specific reaction time, bacteria were then collected by centrifugation for 2 min, washed once and suspended in sterile saline. The bacterial suspension was diluted 10^4^ fold, 10 μL was plating on blood agar plate. After a 24 h incubation period, the bactericidal effects of each solution were determined by observing for bacterial growth in culture [4].

### 2.4. Cell Cultures

Venous blood was collected from healthy adults (25–40 years old). Human peripheral blood mononuclear cells (PBMCs) were isolated from the heparinized venous blood of these donors after centrifugation at 300× g for 20 min over a Ficoll-Hypaque cushion (specific gravity 1.077). Freshly obtained PBMCs were suspended in RPMI-1640 medium (GIBCO, Gaithersburg, MD) containing 10% fetal bovine serum and adjusted to a concentration of 1.0 × 10^6^ cells/mL. Gallium chloride (GaCl_3_) in PBS was then added to final concentrations as 0.01, 0.1, 1, 10, 50, 100 or 1000 μg/mL.

Mucosal mast cell-derived rat basophilic leukemia (RBL-2H3) cell line was purchased from the American Type Culture Collection (ATCC) and grown in Dulbecco’s Modified Eagle Medium (DMEM, Gibco, Grand Island, NY, USA) with 10% fetal bovine serum (FBS) and 100 U/mL penicillin, plus 100 μg/mL streptomycin according to [7]. The cells were maintained in 75 cm^2^ culture dishes at 37 °C in a humidified chamber with 5% CO_2_ in air and were routinely passed at a ratio of 1:2 at least every 4 days until the day of experiment. Prior to the day of experiment, all cells were washed and replenished with fresh culture media.

Neonatal foreskin primary human epidermal melanocytes (HEMn-MP) were purchased from Cascade Biologics™, cultured in Medium 254 (M-254-500; Cascade Biologics™) and supplemented with human melanocyte growth supplement (HMGS, cat. # S-002-5) following [11].

The human melanoma, A375.S2 cell, was cultured in DMEM medium, 10% fetal bovine serum (FBS), 100 U/mL penicillin, 100 μg/mL streptomycin, 0.03% glutamine, and 1 mM sodium pyruvate [13]. The study cells were incubated at 37 °C in a humidified atmosphere containing 5% CO_2_.

### 2.5. Cytotoxicity Assay—Methylthiazoleterazolium (MTT) Assay

The MTT assay was used to determine cell viability and proliferation [9,22]. After seeding cells for 24 h, ACW at concentrations of 2, 5, 10, 20% were added. After 48 h of treatment, MTT were added at the final concentration of 0.5 mg/mL for 2 h of incubation at 37 °C. After 2 h of MTT-treatment, media were removed, and each precipitate in each dish was dissolved in 100 μL of DMSO. After the dishes were gently shaken for 20 min, the OD values of the supernatant were measured at 595 nm.

### 2.6. The Inflammation Evaluation

PBMCs contain physical numbers and ratios of monocytes/macrophages, T cells, B cells and natural killer (NK) cells. Therefore, PBMC was often used in immune regulation tests, and several cell-type-specific activation cytokines in PBMCs were identified [23]. More specifically, we selected TNF-α and IL-4 as monocytes/macrophage triggering cytokines. For the following Th responses, (IFN-γ) was selected as a Th1 activation marker, IL-13 was selected for Th2, TGF-β was selected for Treg, and IL-17A was selected for Th17. Using lipopolysaccharides (LPS) as the inflammation positive control (0.2 μg/mL), we analyzed the selected cytokines by commercially available enzyme-linked immunosorbent assay (ELISA) kits (Quantikine, R&D System, Minneapolis, MN, USA) according to the manufacturer’s instructions. After 48 h of ACW treatments, cultured PBMCs were centrifuged at 1000× g for 10 min and the cell-free supernatants were obtained for cytokine measurements.

### 2.7. The Measurement of De-Granulation

RBL-2H3 cells are tumor analogs of mucosal mast cells and express numerous high affinity IgE receptors, FcɛRI. These cells have been extensively used as a major model for the study of mast cell de-granulation through the antigen-induced aggregation of FcɛRI [7]. Briefly, RBL-2H3 cells were dispensed into a 96-well plate at a density of 1 × 10^5^ cells per well and incubated at 37 °C for 4–6 h to allow their complete adherence onto plate. Subsequently, test ACW were added at different final concentrations (ranging from 0–20%, v/v) and incubation was allowed to continue for 24 h before the addition of mouse anti-DNP IgE (mIgE-DNP). After another 16-h incubation, IgE-sensitized RBL-2H3 cells were washed twice in pre-warmed Tyrode’s buffer (135 mM NaCl, 5 mM KCl, 1.8 mM CaCl_2_, 1.0 mM MgCl_2_, 5.6 mM glucose, 20 mM HEPES, and 1 mg/mL BSA at pH 7.4) and stimulated by adding the cross-linking antigen DNP-BSA diluted in Tyrode’s buffer at 1 μg/mL at 37 °C for 1 h. The reaction was stopped by cooling the plate in an ice bath for 15 min. As controls for the measurement of the total amount and spontaneous release of β-hexosaminidase, unstimulated cells were either lysed with 1% Triton X-100 solution or left untreated (without the addition of antigen), respectively. Aliquots of supernatants collected from the control and experimental wells were transferred into a 96-well microplate and incubated with an equal volume of 1 M p-NAG prepared in 0.1 M citrate buffer (pH 4.5) as substrate at 37 °C for 1 h. The reaction was quenched by the addition of 150 μL stop buffer (0.1 M Na_2_CO_3_/NaHCO_3_, pH 10.0) followed by the measurement of the absorbance at 405 nm on a microplate reader. The inhibition percentage of β-hexosaminidase release from RBL-2H3 cells was calculated using the following equation:

(1) $$\text{Inhibition }(\%)=[1-\frac{\left(\text{ODsample}-\text{ODspontaneous}\right)}{\left(\text{ODtotal}-\text{ODspontaneous}\right)}]\times 100$$

### 2.8. Determination of Anti-Oxidative Properties of ACW *in Vitro*

The cosmetics are particularly suitable for skin care in protecting against oxidative stress and aging phenomena. So, the anti-oxidative exam is a uniform tool for the quality control of raw materials and final products of cosmetics. In this part, the antioxidant analysis methods of ACW include superoxide anion radical scavenging ability, free radical scavenging abilities, ABTS radical scavenging ability, reducing power assay, ferric reducing antioxidant power assay and metal chelating activity assay. The superoxide anion radical scavenging ability was determined according to a modified protocol developed by Wang *et al.* [8]. The PMS (120 μM), NADH (936 μM) and NBT (300 μM) solutions were prepared in 0.1 M sodium phosphate buffer (pH 7.4) and kept on ice for the duration of the experiments. A 1.0 mL aliquot of the extract sample was pipetted into a test tube and diluted with 4.0 mL of 95% ethanol, and then centrifuged at 1000× g and 4 °C for 5 min. One milliliter of supernatant solution was mixed with solutions, followed by the addition of 1.0 mL of PMS, NADH and NBT, respectively. After incubation as a 37 °C thermostatic batch for 5 min in the dark, the absorbance of the reaction mixture was measured at 560 nm against a blank on a spectrophotometer (Hitachi, Model U-2001). The superoxide anion radical scavenging activity (%) was determined as

(2) $$100\times ({\text{OD}}_{\text{control}}-{\text{OD}}_{\text{sample}})/{\text{OD}}_{\text{control}}$$

Most cosmetics and food compounds usually have free radical scavenging abilities. The antioxidant activity of the testing compound was measured in terms of hydrogen donating or radical scavenging ability using the stable DPPH method as modified by [8]. Proper concentrations of the samples were added to 0.2 mL of DPPH (60 μM) solution. When DPPH reacts with an antioxidant compound that donates hydrogen, it is reduced, resulting in a decrease in the absorbance at 520 nm. The absorbance was recorded at 30 min using a UV-visible spectrophotometer. Vitamin C was used as a positive control. The percentages of remaining DPPH were plotted against the sample to obtain the amount of antioxidant required to reduce the initial concentration of DPPH. The scavenging activity calculation formula was similar to Equation (2).

The ABTS radical-scavenging assay was conducted according to the trolox equivalent antioxidant capacity (TEAC) Assay of Damir Iveković [23]. Briefly, each sample solution (0.08 mL) was mixed with 4.92 mL of the ABTS solution (7 mM final concentration in 5 mM phosphate buffer (pH 7.4)). The test solution was mixed for 30 s, and the absorbance at 734 nm was measured at 30 °C after precisely 10 min. The appropriate blank solvents were run in each assay, and the percentage inhibition was calculated for the blank absorbance at 734 nm. The activity of a sample was expressed as microgram equivalents of trolox per milligram.

A method developed by Oyaizu [24] for reducing the power test was used. The above samples (2.0 mL) and BHT methanolic solutions were spiked with 2.0 mL of phosphate buffer (0.2 M, pH 6.6) and 2.0 mL of 1% potassium ferricyanide. The mixture was then kept in a 50 °C water-bath for 20 min. The resulting solution was then cooled rapidly, spiked with 2.0 mL of 10% trichloroacetic acid, and centrifuged at 3000 rpm for 10 min. The supernatant (2 mL) was then mixed with 2 mL of distilled water and 0.4 mL of 0.1% ferric chloride. The absorbance at 700 nm was then detected after reaction for 10 min. The data were expressed as milligram BHT.

The ferric reducing antioxidant power (FRAP) was determined using a modification assay described by Wang *et al.*, 2010 [8]. The FRAP reagent was prepared from a 300 mM acetate buffer (pH 3.6), 20 mM ferric chloride and 10 mM TPTZ in 40 mM hydrochloric acid. All three solutions were mixed together in the ratio of 10:1:1 (v/v/v). The FRAP assay was performed using reagents preheated to 37 °C. Aliquots (1.0 mL) of the extract samples were transferred into the tubes and diluted with 4 mL of 95% ethanol, and then centrifuged at 1000× g and 4 °C for 5 min. 0.1 mL of supernatant solution was mixed with 3.0 mL of FRAP reagent and 0.3 mL of deionized water. After incubation as a 37 °C thermostatic batch for 30 min in the dark, the absorbance of the reaction mixture was measured at 593 nm against a blank containing 3.0 mL of FRAP reagent and 0.4 mL of de-ionized water on a spectrophotometer (Hitachi, Model U-2001). Ferrous sulfate heptahydrate solution was used to perform the calibration curves. The data were expressed as milligram ferrous sulfate heptahydrate.

The ferrous ion-chelating potential of chlorophyll was investigated according to the method described by Wang *et al.*, 2010 [25]. Testing samples at suitable concentrations dissolved in DMSO were added to a solution of 2.0 mM FeCl_2_·4H_2_O (0.05 mL). The reaction was initiated by the addition of 5 mM ferrozine (0.2 mL) and the mixture was vigorously shaken and left standing at room temperature for 10 min. After the mixture reached equilibrium, the absorbance of the mixture was measured at 560 nm against a blank. EDTA was used as a positive control and chelating activity calculation formula was similar to Equation (2).

### 2.9. Mass Spectrometry Analysis

ACW was analyzed by Finnigan TSQ Quantum Ultra tandem triple quadrupole mass spectrometry (Thermo Fisher Scientific, San Jose, CA, USA). A syringe pump sets up a flow rate at 1 μL/min to separate sample into a NanoSpray ion source of mass spectrometry. The acidic water sample was analyzed in the positive mode with 1800 V spray voltage, and scan *m/z* range was 20–135.

### 2.10. Cellular Tyrosinase Activity Assay and Melanin Quantification Examination

In human skin melanocytes, tyrosinase inhibition abilities of the ACW were examined by the conversion of L-tyrosine and oxidation of L-dopa to dopaquinone. Cellular tyrosinase activity assay was based on the method [11] with some modifications. HEMn-MP (105 per well) placed in 24-well micro-plate in 500 μL of medium containing various concentrations of testing samples were incubated for 2 days. The sample-treated cells were washed with PBS and lysed with 0.5% Triton X-100/PBS. The lysate was mixed by vibration with 10 μL of 2 mM L-tyrosine in 0.1 M phosphate buffer (pH 6.8). After incubation at 37 °C for 3 h, the absorbance at 490 nm was measured on a spectrophotometer.

Cells were incubated in 6 well-plates at a density of 5 × 10^5^ cells per well to study the melanin content. Cell pellets were dissolved in 2.0 N NaOH containing 10% DMSO and heated at 80 °C for 1 h and suspensions were clarified by centrifugation for 10 min at 10,000 g. The amount of melanin was determined spectrophotometrically, based on the absorbance at 475 nm.

### 2.11. The Assessment of Cell Migration

Th determination of cell migration ability was described previously [26]. In brief, 1 × 10^5^ cells were seeded onto 12-well plates, treated with ACW or DMSO, and then grown to complete confluence. A yellow 200 μL plastic pipette tip was used to create a clean 1-mm-wide wound area in the A375.S2 confluent culture. After incubation for 24 h and 48 h, the wound gaps were photographed. The wound areas were then examined and calculated using the software “TScratch” [26].

### 2.12. Statistical Analysis

All results are expressed as the mean values with ± standard deviation (SD). Statistical comparisons were carried out using the Student’s *t*-test for paired values.

## 3. Results and Discussion

### 3.1. Antimicrobial Activity against *E. coli* and *S. aureus*

In our current study, we investigated the anti-microorganism potential of ACW by testing *S. aureus* and *E. coli* growth. For the test validity, the growth control well should always be positive and the negative control and reagent control wells should always be negative. ACW showed the satisfactory activity against gram-positive bacteria, *S. aureus*. The results of this antibacterial assay were shown in Table 1. It inhibited 95% growth of *S. aureus* after 30 s of exposure. The pH of ACW was 3.35 measured by pH meter. The survival data of gram-negative *E. coli* indicated that the bactericidal effect was less significant than on the *S. aureus*. A longer incubation time (5 min) was required to inhibit 52% of bacteria growth.

### 3.2. Anti-Inflammation Measurements of ACW

**Figure 2:**
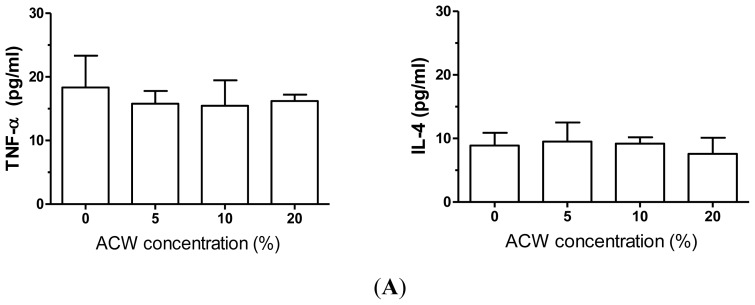

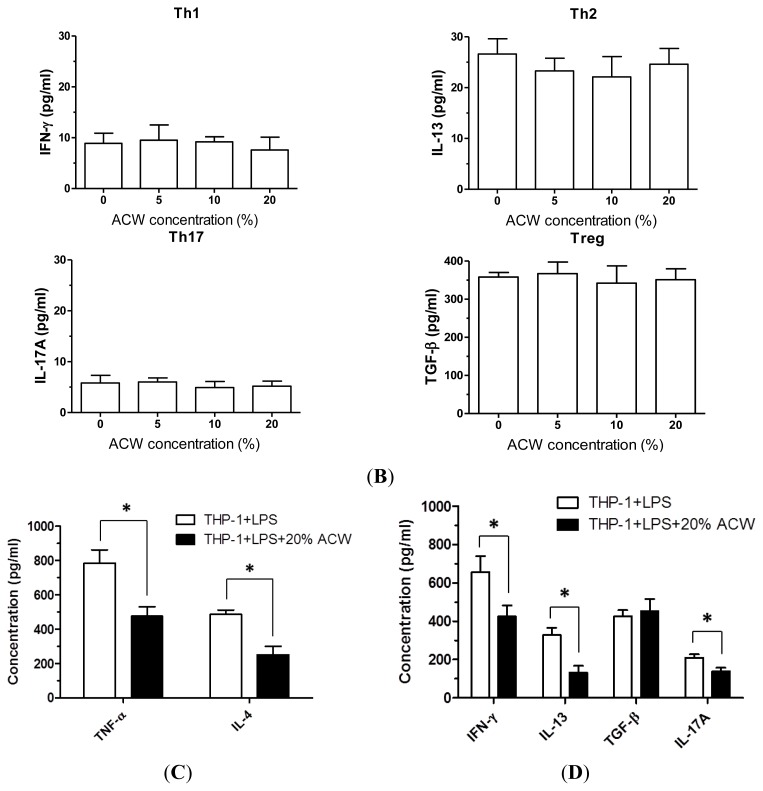
(**A**) Monocyte/macrophage cytokines released from PBMCs treated with ACW and measured by ELISA; (**B**) Th cell type-specific cytokines released from ACW treated PBMCs by ELISA; (**C**) Effects of ACW on monocyte/macrophage cytokines released from LPS-stimulated PBMCs; (**D**) Effects of ACW on Th cytokines released from LPS-stimulated PBMCs. * *p* < 0.05 tested *vs.* control; mean ± SD.

Since ACW showed a significant anti-bacteria effect, we further tested several possible side effects of ACW, *i.e.*, inflammatory and allergic effects. Without LPS stimulation, 5%, 10% or 20% of ACW treatments did not induce significant changes in triggering cytokines TNF-α or IL-4 releases from cultured PBMCs after 48 h of treatments (Figure 2A). Similarly, ACW treatments did not considerably affect IFN-γ, IL-13, TGF-α or IL-17A levels (Figure 2B). However, with LPS (0.2 μg/mL), the bacteria endotoxin eliciting strong inflammatory responses, were treated during cell culture; 20% of ACW showed significant effects on cytokine levels. In our PBMC culture model, LPS extensively increased TNF-α, IL-4, IFN-γ, IL-13, and IL-17A concentrations, but not TGF-β. ACW (20%) significantly decreased the LPS-induced TNF-α and IL-4 release from PBMCs, showing an inhibitory effect on monocyte/macrophage activation (Figure 2C). The secretion of Th1 activation marker IFN-γ and Th2 activation marker IL-3 were blocked by ACW under LPS stimulation. ACW also presented an obvious inhibitory effect on LPS-induced IL-17A release (Th17 marker), but not in Treg associated TGF-α levels (Figure 2D).

Our data indicated that ACW treatments did not have stimulatory effects on cytokine releases in untreated normal human PBMCs. However, during an activated inflammation condition by LPS, ACW showed significant inhibitory effects. Since the TNF-α release from monocytes/macrophages regulated the Th1-mediated inflammatory responses, short-term nontoxic dosage treatment of ACW was possible to be used in inflammatory lesions. For example, it might benefit insect bite lesions or infectious wounds. In addition, the ACW-induced decrease in Th2 cytokine (IL-13) might be useful for certain allergic disease conditions, such as atopic dermatitis. In contrast, ACW did not show a significant inhibitory effect in Treg response (TGF-β) release. Although the TGF-β is known to induce toxic effects in epithelial cells, including epidermal keratinocytes, a sufficient level of TGF is required for fibroblast proliferation, such as wound healing conditions. Therefore, ACW has a potential to be used in human epidermal related products, with the advantage of an anti-inflammatory effect, but without the adverse effect in fibroblast regeneration.

### 3.3. Allergy-Free Test on ACW

Among the various inflammatory mediators produced by mast cells, β-hexosaminidase is stored in the secretory granules of the cells and is released by exocytosis when mast cells are immunologically activated, such as the cross-linking of FcɛRIs. Therefore, the measurement of an inhibitory capacity on the released β-hexosaminidase has been commonly used as a reliable parameter to predict possible anti-allergic activities of either natural or synthetic compounds.

**Figure 3:**
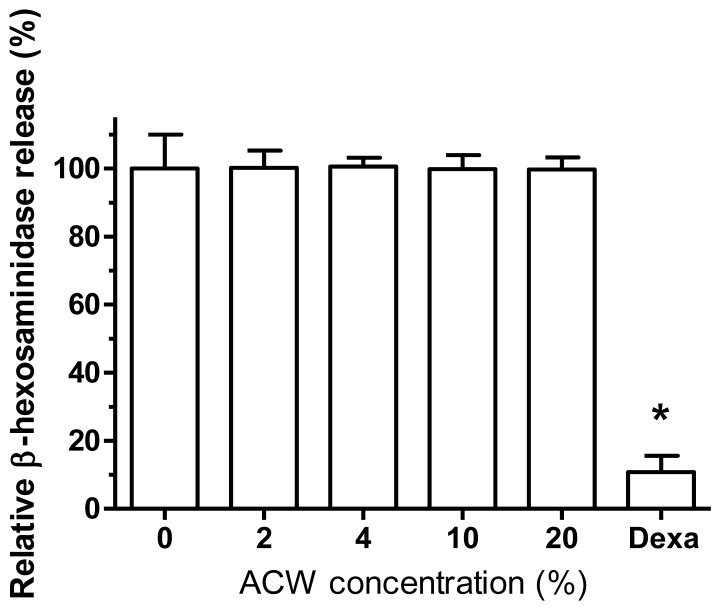
ACW inhibitory effects on β-hexosaminidase release from antigen-stimulated RBL-2H3 cells. Dexamethasone (Dexa) was used as a positive control. * *p* < 0.05 tested *vs.* control; mean ± SD.

De-granulation is one of the cardinal features of activated mast cells or basophils upon the stimulation of cross-linking antigens [27]. To assess the possible modulatory effects of ACW on the activation of RBL-2H3 cells, test samples incubated with RBL-2H3 mast cells for 24 h before the combination of mIgE-DNP and DNP-BSA were added to trigger the activation of the cells. The inhibitory capacity of ACW was evaluated as the release of β-hexosaminidase in the culture media as compared to those from IgE-activated cells. As shown in Figure 3, RBL-2H3 pre-treated with dexamethasone (10 nM) was included as a positive control to exhibit significant inhibition. None of the various concentration samples exhibited extra release of β-hexosaminidase from activated RBL-2H3 cells to signify allergy-free property of ACW.

### 3.4. Antioxidant Capacity of ACW

The following purpose of this section was to survey the antioxidant properties of ACW and the test samples were added at various concentrations (ranging from 0–20%, v/v). The radical scavenging activities were investigated by: scavenging of DPPH, ABTS and superoxide anion-free radicals; reducing power was demonstrated by iron ions reduction reaction; FRAP was demonstrated by Fe(III)-TPTZ complex ions reduction reaction; and metal chelating power was completed by ferrous ions. Superoxide anion radicals are generated in a PMS-NADH system by oxidation of NADH. The color variation between superoxide anion radicals and NBT was measured at 560 nm. The decrease of absorbance at 560 nm indicated the consumption of superoxide anion in the PMS-NADH-NBT system to present the strong scavenging from ACW (Table 2). Further investigations will focus on this part in the future. No visible radical scavenging capacities were detected from ACW on DPPH or ABTS systems. The radical scavenging activity of against superoxide anion radical was evaluated by the reduction of NBT. ACW induced the reduction of the Fe^3+^/ferricyanide complex to the ferrous form with minor effects. The FRAP or metal chelating methods did not demonstrate anti-oxidative properties from ACW.

### 3.5. Mass Spectrometry Analysis

For water quality analysis, induced couple plasma mass spectrometry (ICP-MS) is a popular MS detected technique, especially in toxic heavy metal ions in which these metal ions were not observed, such as hexavalent chromium (Cr^6+^), mercury ion (Hg^2+^), cadmium (Cd), lead (Pt) and arsenic (As) and organic metals such as methyl mercury (Hg(CH_3_)^+^) and dimethyl arsine hydroxide (HOAs(CH_3_)_2_). Therefore, MS is considered a sensitive instrument for water quality analysis due to different isotopes. ACW was originally fabricated by TAIYEN Co., which retained effective minerals such as magnesium ion (Mg^2+^), calcium ion, sodium ion and potassium ion, excluding heavy metal ions.

**Figure 4:**
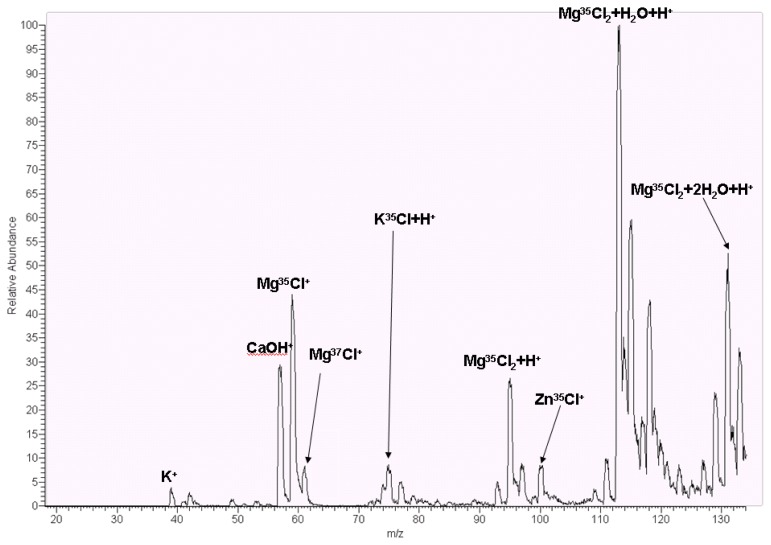
ESI-MS spectrum of ACW in which various metal ions are observed.

Herein, we utilized liquid chromatography mass spectrometry (LCMS) for ACW water analysis. In the data (Figure 4), potassium ion (K^+^) with *m/z* (mass charge ratio) 39 was observed, and potassium chloride with proton (KCl + H^+^) with *m/z* at 75 Da. Inflammation inhibitor factors such as magnesium and zinc ions, Mg^2+^ ion couples with one chloride ion to generate *m/z* at 59 Da, and chloride ion with isotope 37 Da generates with Mg^2+^ to compose at 61 Da. Magnesium ion generated two chloride ion to compose Mg^35^Cl_2_, Mg^35^Cl^37^Cl, and Mg^37^Cl_2_ ion which hydrated with proton ion (H^+^), and to acquire a mass/charge ratio with 95 Da, 97 Da, and 99 Da. Due to concentration of ^37^Cl, it was 25% in nature, *m/z* 99 Da and Mg^37^Cl_2_ had low abundant intensity. In MS, metal ions produced with water to compose hydrated micelles, such as *m/z* 113 Da and 131 Da coupling, with one and two hydrogen oxides which are generated (Mg^35^Cl_2_ + H_2_O + H^+^) and (Mg^35^Cl_2_ + 2 H_2_O + H^+^). One of the other metal ions was zinc ion which was generated with chloride to compose *m/z* 100 Da. The residue metal ion, such as calcium ion, is *m/z* 57 Da. The results obtained in MS instrument demonstrated the effects of anti-inflammation which might be displayed by magnesium ions. In the previous study, Mg^2+^ is a remission agent for inflammatory skin [28]. The other metal ion, zinc, in the previous study, zinc sulfate solution reduces inflammation in colitis in rats [29]. However, zinc ions provoked anti-inflammation and pro-inflammation depending on intracellular free zinc ion concentration [30]. As shown in the MS spectrum, there was no free zinc ion, and zinc ion coupling with chloride was revealed to be low intensity as well as ACW was utilized on skin as a cosmetic.

### 3.6. Skin-Whitening Examination on ACW

**Figure 5:**
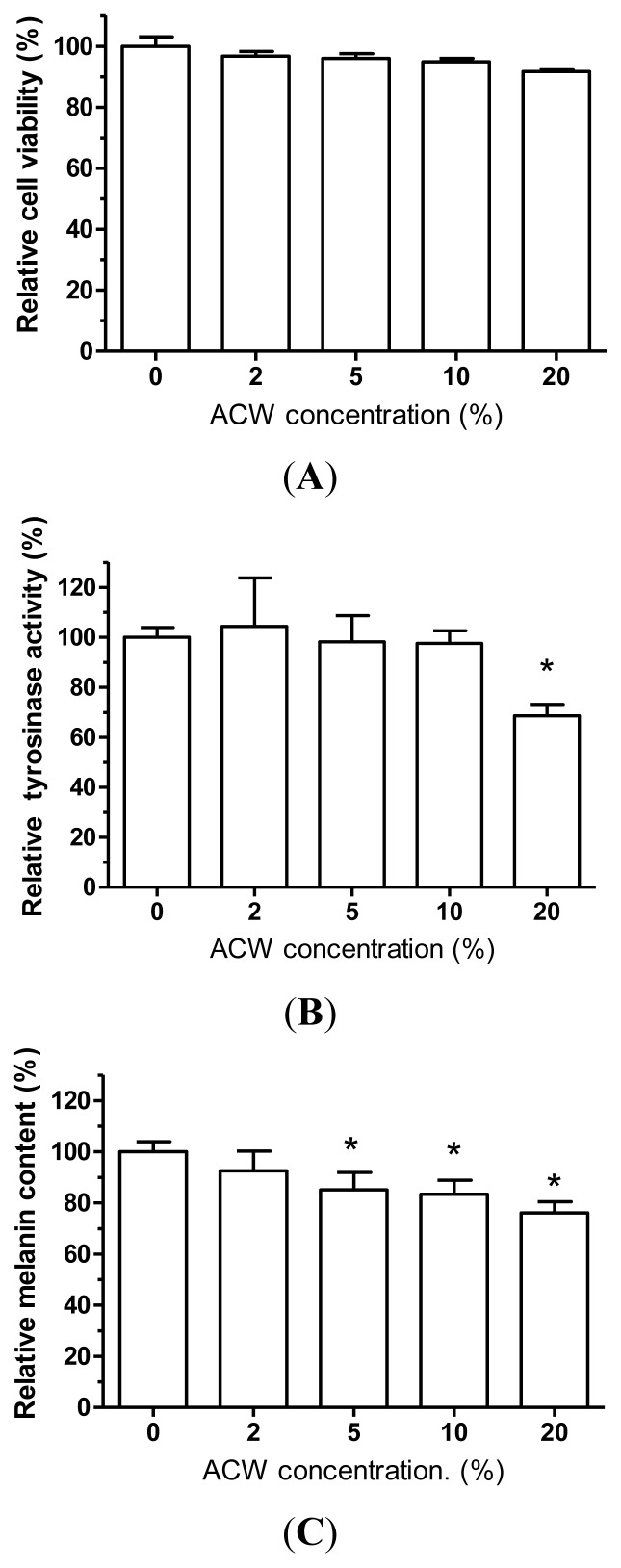
(**A**) The cell viability of human normal skin melanocytes after treated with ACW, tested with MTT assay. (**B**) The tyrosinase activity of HEMn-MP after treated with ACW. (**C**) The tyrosinase activity of HEMn-MP after treated with ACW. * *p* < 0.05 tested *vs.* control; mean ± SD.

As a potent skin lightening agent, ingredients should be harmless, without undesirable cytotoxic side effects. MTT assay was used to measure cell viability and investigate whether ACW would induce cell death adversely. ACW were treated with various concentrations from 0 to 20% to verify the dose-dependent effects. In Figure 5A, human normal skin melanocytes exposed to high testing concentration (20%) exhibited viabilities of more than 85% after 48 h of treatment. Overall, there was no significant toxicity with the testing conditions, and this result revealed that ACW had very little discernible toxicity to human epidermal cells.

The inhibition of tyrosinase activity was described in numerous reports, most of which, however, used mushroom tyrosinase as the model. Mushroom tyrosinases have significant differences from human tyrosinases in catalysis mechanisms [8,31–33]. We have known that the catalysis of the human tyrosinase and mushroom tyrosinase have diverse mechanism reactions. As to mammalian skin color, tyrosinase activity and melanin are two important factors. With L-DOPA as the substrate, melanin productions by the two types of enzymes have separate solubility properties, and L-DOPA has no effect upon mammalian enzyme activities but would stimulate the mushroom enzyme [11]. ACW was investigated for human cellular tyrosinase-inhibiting abilities and melanin content decreasing powers in human melanocytes. With increasing concentrations of ACW, the human tyrosinase activities and melanin contents were decreased (Figure 5B). ACW demonstrated the highest inhibition at a dosage of 20% to tyrosinase activities and melanin contents (Figure 5C). Besides, in Figure 5B,C, the melanin contents matched with the tyrosinase activities in the same dose-dependent tendencies upon ACW, which demonstrated that the epidermal cellular melanin reductions might be due to the inhibition of human tyrosinase activities.

### 3.7. ACW Attenuated A375.S2 Melanoma Proliferation and Migration

**Figure 6:**
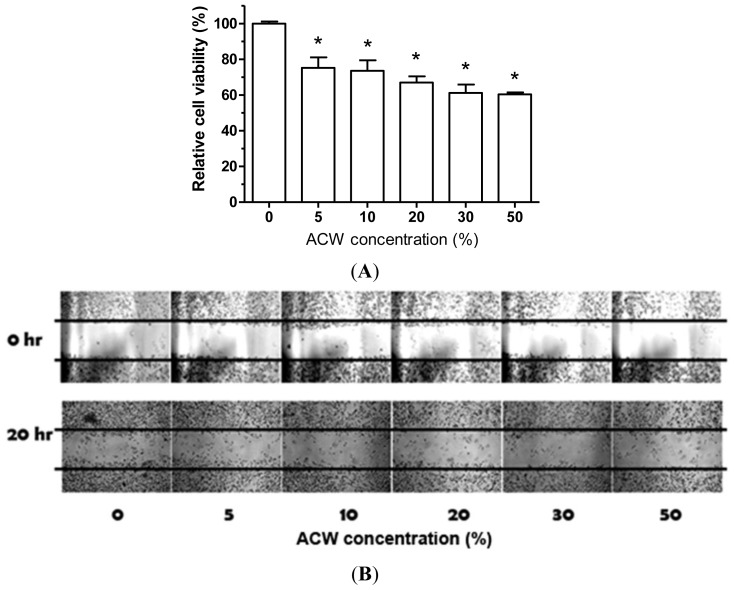
(A) Proliferation of A375.S2 cells is inhibited by ACW. Cells were incubated with indicated percentages of ACW for 24 h. The proliferation was determined by MTT assay. (**B**) ACW inhibits cell migration in A375.S2 cells. A total of 5 × 10^5^ cells were seeded onto a 12-well plate and the cells were scraped to create a clean 1-mm-wide wound area. Cells were treated with the indicated percentages of ACW for 16 h. The wound gaps were photographed and analyzed using the software TScratch. * *p* < 0.05 tested *vs*. control; mean ± SD.

There is no literature that reports the biological effects of ACW on cancer cells. Therefore, we examined whether ACW could have advantages on human skin melanoma cells. As shown in Figure 6A, the proliferation of A375.S2 cells was observed to attenuate by ACW from 5 to 20%. Reactive oxygen species (ROS) such as superoxide O^2−^, was reported to serve as a second messenger for signaling amplification [34]. Moreover, ROS were shown to be essential for cellular proliferation and migration [35–37]. A higher intrisic ROS level was detected in cancer cells and has shown to be correlated with cancer metastasis [38]. Therefore, many anti-cancer strategies were based on modulating the intracellular ROS of cancer cells [39,40]. As shown in Table 1, ACW dramatically reduced the level of superoxide, indicating the ROS scavenging potential of ACW. The result of wound healing assay showed that the cell migration of A375.S2 cells was significantly inhibited by ACW in a dose-dependent manner (Figure 6B).

## 4. Conclusion

The present study aimed to examine the biological properties of ACW. By testing multiple biological effects, a significant anti-bacterial effect was identified. ACW had bactericidal effects against *S. aureus*, but little on *E. coli*. Based on our present screening, this anti-bacterial effect of ACW did not show adverse effects to the immune system. As the bio-assay data, ACW showed an anti-inflammation outcome, especially in suppressing bacterial LPS stimulated TNF-α release. Furthermore, our study demonstrated that various concentrations of ACW exhibit no release of allergy mediator. Since increased TNF-α is known to be associated with many pathological conditions, such as infection/inflammation, psoriasis, as well as cancers, the inhibitory effect of ACW on TNF-α potentially has a wild-range of applications. Anti-oxidative properties, including free radical scavenging, metal chelating and reducing power were found to be minor effects compared with the positive controls. Interestingly, ACW possessed a high superoxide anion radical scavenging aptitude. The mass spectrometry data identified that ACW contained high levels of magnesium, zinc, potassium and calcium ions, and might provide possible explanations for these immune-regulatory effects. ACW as a melanogenic regulatory agent illustrated potential safety without the decrease in human skin cell viabilities for skin whitening. More importantly, to our best knowledge, this is the first demonstration that ACW dramatically attenuates the migration ability of cancer cells. The data exhibited the high potential of applying ACW in medical cosmetology, food supplementation, antibiotics or chemotherapy. Long-term toxic tests and ion-specific effects are needed for further applications.

## Figures and Tables

**Table 1:** Comparison of colony count (CFU per milliliter) results of *S. aureus* and *E. coli* incubated at the ACW for 5, 30, 60, 180, 300 and 900 s.

ACW	Colony Count (×10^6^ CFU/mL) (% Bacterial Growth)						

	Control	Exposure Time (s)					

		5	30	60	180	300	900
*S. aureus* (29,213)	272	30 (11%)	14 (5%)	18 (7%)	43 (16%)	50 (18%)	23 (8%)
*E. coli* (35,218)	243	159 (65%)	180 (74%)	212 (87%)	191 (79%)	150 (62%)	117 (48%)

**Table 2:** Various antioxidant effects of ACW.

ACW	Superoxide Anion Scavenging (%)	DPPH^·^ Scavenging (%)	ABTS^+^ Scavenging (%)	Reducing Power c (OD_700_)	FRAP Assay (FeSO_4_ mg/mL)	Metal Chelating Activity (%)
Gallic acid a	100 ± 0.00	100 ± 0.00	100 ± 0.00	0.284 ± 0.04	10.31 ± 0.23	–
EDTA b	–	–	–	–	–	100 ± 0.00
2%	93.97 ± 0.25	nd	nd	0.044 ± 0.05	nd	nd
5%	95.88 ± 0.39	nd	nd	0.044 ± 0.08	nd	nd
10%	96.47 ± 0.49	<10.0	nd	0.045 ± 0.01	<1.0	<10.0
20%	96.86 ± 0.10	<10.0	nd	0.043 ± 0.05	<1.0	<10.0

a Gallic acid was used as a positive control at 0.5 mg/mL;

b EDTA was used as a positive control on metal chelating ability at 100 μM;

c Absorbance was measured at 700 nm;

(−) is no testing; nd: not detectable. Data were expressed as a mean value of at least three independent experiments.
